# Subacute Urinary Retention due to a Subpubic Cartilaginous Cyst Treated with Surgical Resection and Internal Fixation: A Case Report and Review of the Literature

**DOI:** 10.1155/2018/5736341

**Published:** 2018-01-14

**Authors:** Yu Taniguchi, Hiroshi Kamada, Hisashi Sugaya, Tomofumi Nishino, Hajime Mishima, Naoyuki Ochiai, Masashi Yamazaki

**Affiliations:** ^1^Department of Orthopaedics Surgery, Faculty of Medicine, University of Tsukuba, 1-1-1 Tennodai, Tsukuba, Ibaraki 305-8575, Japan; ^2^Division of Regenerative Medicine for Musculoskeletal System, Faculty of Medicine, University of Tsukuba, 1-1-1 Tennodai, Tsukuba, Ibaraki 305-8575, Japan

## Abstract

A subpubic cartilaginous cyst is a rare mass lesion derived from the pubic symphysis, which can cause acute or subacute urinary retention. We report a case of a subpubic cartilaginous cyst in a 62-year-old woman that caused lower abdominal pain and subacute urinary retention, requiring surgical resection. On physical examination, a hard, flexible, nontender mass, 4 cm in diameter, was palpable along the lower border of the pubic bone, extending to the perineum. Magnetic resonance imaging revealed a clearly distinct (3.8 cm × 3.8 cm × 7.2 cm) mass on the midpelvic side of the pelvis, centered on the pubic joint. We proceeded with en bloc resection of the mass, including a resection margin of 1 cm on either side. The bony defect was fixed with a locking plate. On pathological assessment, the mass was diagnosed as a subpubic cartilaginous cyst arising from the cartilage of the pubic symphysis. No tumor recurrence was identified over a 4-year follow-up. Based on our experience, we propose that en bloc resection of the mass, including a wider resection centered on the pubic symphysis, with internal fixation, is a possible treatment for a subpubic cartilaginous cystic mass lesion.

## 1. Introduction

A subpubic cartilaginous cyst is a rare soft-tissue mass lesion derived from the pubic symphysis, which can be either asymptomatic or cause discomfort, pain, and urinary retention. Here, we describe a case of a subpubic cartilaginous cyst causing subacute urinary retention, which required surgical treatment. We also provide a review of available literature on this clinical condition.

## 2. Case Presentation

A 62-year-old woman presented with a chief complaint of lower abdominal pain and urinary retention. Her relevant past medical history included three pregnancies and deliveries, and a hysterectomy and removal of ovaries, bilaterally, due to endometriosis at the age of 32 years, with subsequent menopause. Lower abdominal pain and urinary obstruction appeared 5 months prior to hospitalization. At that time, the patient visited another hospital and was treated with catheterization. Following the initial catheterization, she was unable to void and was recatheterized. Magnetic resonance imaging (MRI) revealed a mass between the pubis and the urethra, and the patient was referred to our hospital for assessment and treatment.

**Figure 1:**
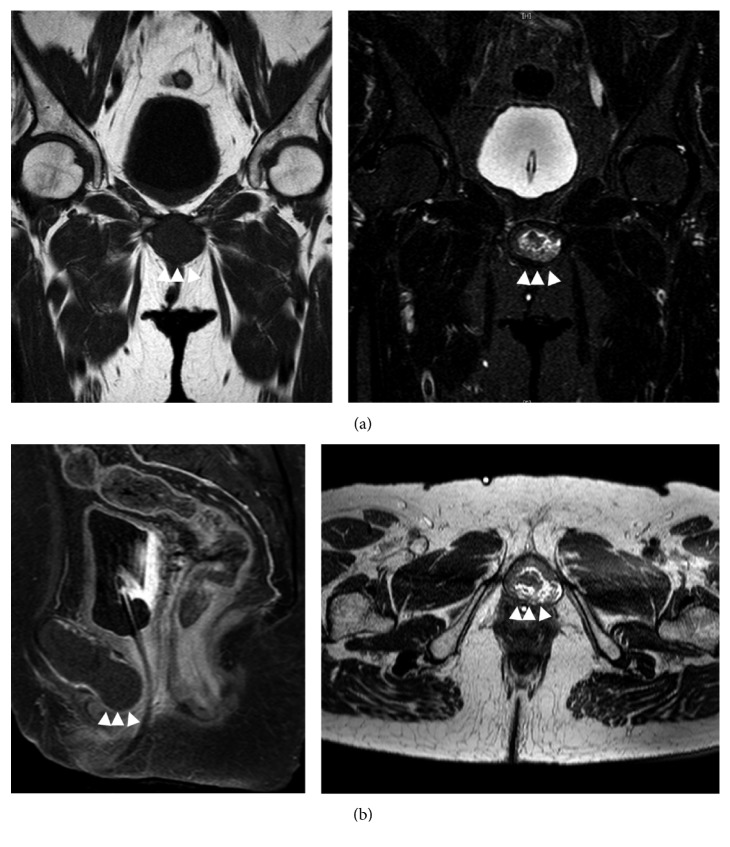
Magnetic resonance imaging (MRI) findings. (a) Coronal plane, noncontrast, T1-weighted, STIR images (△△△, subpubic cyst). (b) Sagittal plane, gadolinium-enhanced, T1-weighted image, showing compression of the ureter from the anterior, and axial plane T2-weighted image.

On physical examination, the hard and flexible tumor was palpable, about 4 cm in length, extending from the lower side of the pubic bone to the perineum, but with no tenderness. On gynecological examination, the external urethral meatus was deviated posteriorly, but with no deformation. A needle biopsy was performed, with the histological diagnosis being difficult to judge owing to the few cell components available in the biopsy specimen. Urinary cytology was assessed as class II. Magnetic resonance (MR) imaging revealed a clearly distinct midpelvic mass lesion (3.8 cm × 3.8 cm × 7.2 cm), centered on the pubic symphysis. T1-weighted imaging revealed well-defined borders of the lesion, with a low signal intensity inside. On the short-TI inversion recovery (STIR) sequence, the inside of the mass appeared mottled, with mixed low and high intensity signals. After intravenous administration of gadolinium, the mass lesion showed contrast enhancement in the marginal region, but with no contrast enhancement in its internal compartment. A urethral catheter was visible on the posterior aspect of the mass lesion, which had been placed for treatment of the acute urinary retention disorder (Figure 1).

Based on consultation with urology and gynecology, the mass was deemed to be nonmalignant, arising for the pubic symphysis. As the mass was causing urinary symptoms, a decision was made in consultation with the patient for surgical resection.

**Figure 2:**
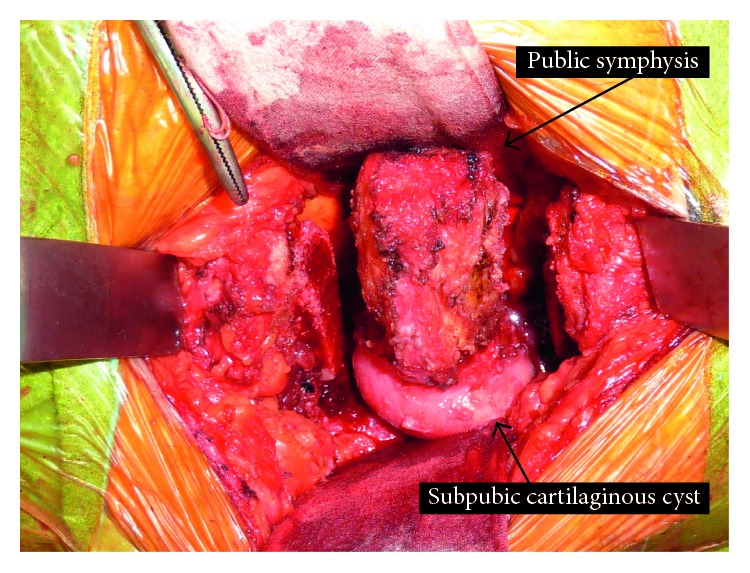
An intraoperative photograph after bilateral pelvis osteotomy for excision of the subpubic cartilaginous cyst, including the pubic symphysis.

Surgical resection was performed in the crushed stone position. The anterior surface of the pubis was exposed over an arc of about 15 cm, the subcutaneous tissue was expanded, and the pubic symphysis exposed as a whole. The soft tissues adhering to the pubic bone, such as ligaments, were peeled off, at both the anterior and posterior surfaces, and the mass was confirmed. The mass was excised en bloc, including the pubic symphysis, using an osteotomy at 1 cm to the left and right of the pubic symphysis. After the 2 cm en bloc resection, the remnants of the pubic bone separated further, to about 4 cm (Figure 2). About 3 cm of the tricortical bone was collected from the iliac bone and inserted between the pubic bones to close the gap, and it was fixed with a 7-hole locking plate and 6 screws.

**Figure 3:**
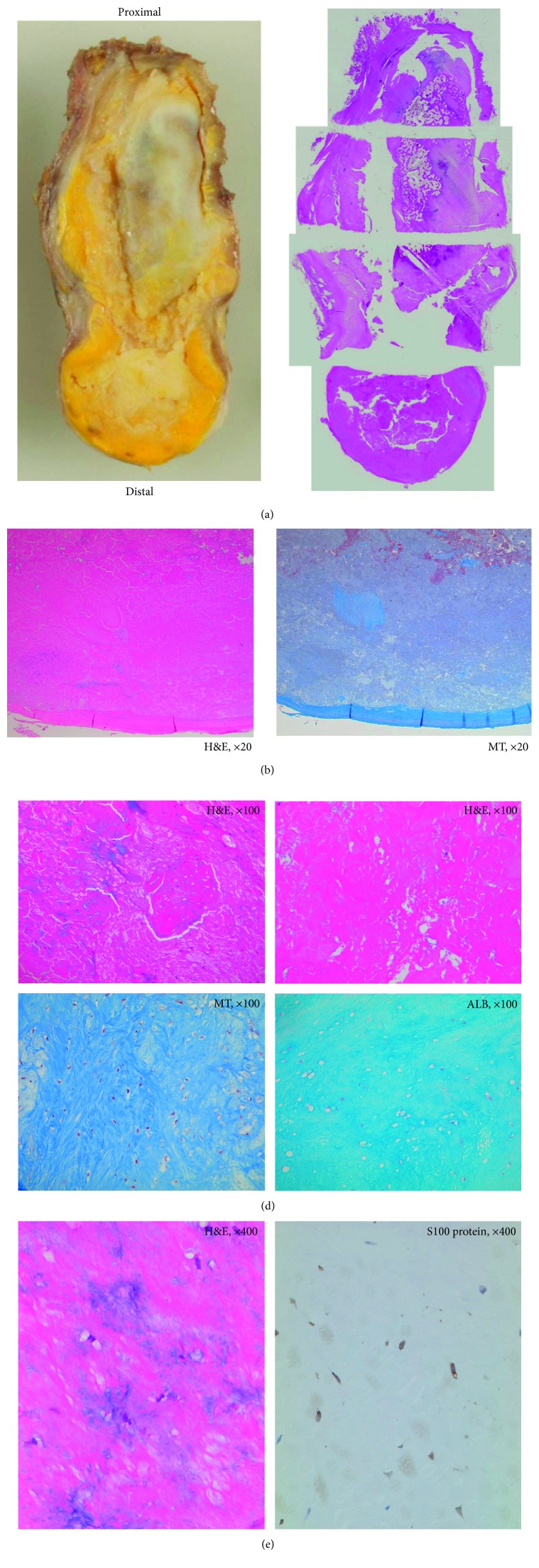
Pathological findings. (a) Macrophotography of the pubic symphysis and subpubic cyst; the whole image of the microphotograph is shown. (b) From the capsule to the margin of the mass, Masson trichrome (MT) staining identified fibrocartilage as the main tissue. (c) Microscopic section (magnification 100x) of the center of the cyst, with MT staining revealing denatured fibrocartilage, with a fiber-rich section. Alcian blue (ALB) staining revealed an abundant mucous substance, which included a modified product derived from the cartilage. (d) Microscopic section (magnification 400x) of the center of the cyst, showing positive staining of the cartilage tissue using S100 protein immunohistochemistry.

Pathological findings of the mass revealed a cystoid tissue organization. The connective tissue of the wall of the cyst was continuous with the fibrous cartilage of the interpubic disc. Most of the yellowish tissue inside the tumor and around the interpubic disc consisted of denatured acidophilic and ineffective tissues, with poor cellular components, as well as cartilage tissues identified using S100 protein immunohistochemistry. The yellowish tissue was confirmed to be the degenerated fibrocartilage tissue, with no evidence of malignancy (Figure 3).

**Figure 4:**
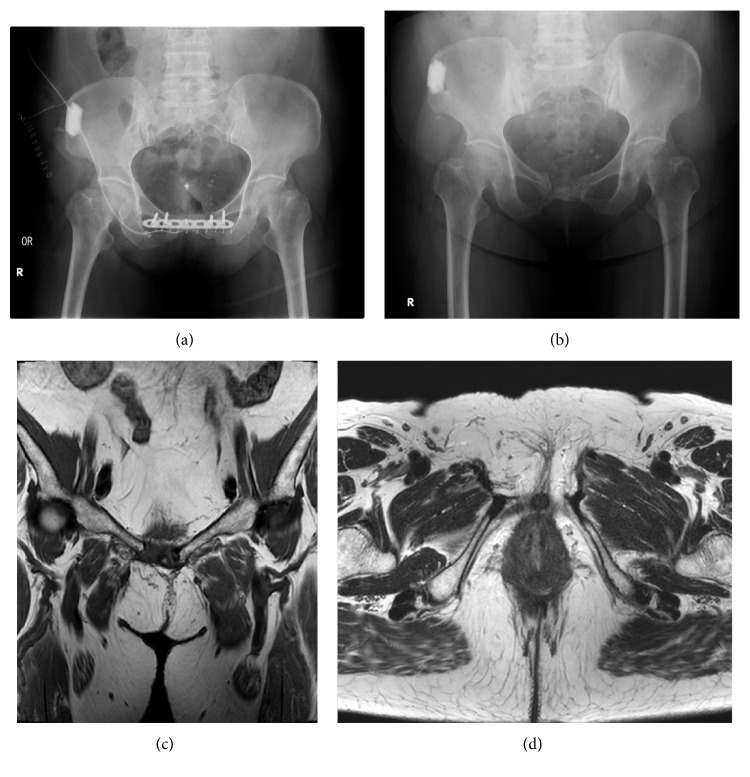
Postoperative standing radiographs (anterior-posterior view) of the pelvis and hips, obtained immediately after surgery (a) and at 4 years postoperatively (b). (c) and (d) Magnetic resonance images obtained at 4 years postoperatively. There is no evidence of tumor recurrence.

The urethral catheter was removed two weeks after surgery, with the patient being able to excrete urine without residual volume or incontinence. The plate with anchored fixation was loosened and removed about 3 months after the surgery. At the 4-year follow-up, urinary function remained normal, with no evidence of recurrence of the mass on MR imaging (Figure 4).

## 3. Discussion

A subpubic cartilaginous cyst is a rare mass derived from the pubic symphysis. The first two cases were reported by Alguacil-Garcia and Littman in 1996 [1], with 18 cases having subsequently been reported [2–16]. A summary of these cases is provided in Table 1. Among these cases, patients ranged in age from 54 to 75 years (2 males and 16 females), with all female patients being multipara. Previous histological assessment of these masses reported the presence of the degenerated fibrocartilage in the central part of the mass, which is consistent with our findings. The pubic symphysis is an anatomically hemiarthrotic joint, composed of a fibrocartilaginous interpubic disc sandwiched between thin hyaline cartilages [17], which is consistent with the fibrocartilaginous nature of the subpubic mass.

Among the 18 cases previously reported, the mass presented as a painless lump in 6 cases, with the other female patients reporting a feeling of discomfort in the vulva and pain with activities of daily living. Urinary symptoms were reported in 5 of these 18 cases, consisting of incomplete bladder emptying, with difficult micturition in 4 cases and acute urinary retention in the other case. In our case, the patient experienced subacute urinary retention requiring indwelling of a urinary catheter. As in the case presented by Ghareeb et al. [15], in our patient, the mass was large and was growing on the posterior aspect of the pubic symphysis, physically occluding the urethra.

As subpubic masses are rare, there is no standard treatment. From the available literature, 7 asymptomatic cases were followed up by observation, with no evidence of a change in the size of the mass among 5 patients over a follow-up period of 2 to 4 years. The 11 cases with urinary tract symptoms, as in our case, underwent surgical resection of the tumor, with no recurrence over a follow-up period of 1 to 3 years. Hoogendoorn et al. [8] reported pubic symphysis dehiscence as a complication of resection of the subpubic cartilaginous cyst. Internal fixation is recommended in cases of pubic symphysis dehiscence if conservative therapy, including reduction in activity and use of a pelvic belt, does not improve symptoms. Due to loosening, we removed the fixation plate early in the postoperative course, at 3 months after surgery, without complication and achieving good bone fusion. Based on our experience, we propose that en bloc resection of the mass, with a wider resection centered on the pubic symphysis, with internal fixation, is a possible treatment for a subpubic cartilaginous cystic mass.

## Figures and Tables

**Table 1:** 

Author, year	Age	Sex	Symptoms	Size	Treatment	Progression after operation
Alguacil-Garcia and Littman, 1996 [1]	61	Female	Groin pain and paresthesia over the medial thigh	20 mm	Surgical	No recurrence over a 3-year follow-up
	58	Female	Dyspareunia and difficulty initiating urination	40–50 mm	Surgical	No recurrence over a 1-year follow-up
Kim and Beasley, 2004 [12]	70	Female	Painless lump	40 × 40 mm	Surgical	N/A
Martel and Spouge, 2007 [10]	72	Male	No symptom	3 cm diameter	Observation	Size reduction (on MRI) after 6 months
Ergun et al., 2008 [13]	54	Female	Chronic abdominal pain	N/A	Observation after needle biopsy	No change (on MRI) over a 2-year follow-up
Judson et al., 2009 [16]	62	Female	Painful vulvar mass	18 × 10 × 12 mm	Surgical	No recurrence over a 2-year follow-up
Bullock et al., 2009 [9]	—	Female	Painless lump	38 mm	Observation	No change over a 2-year follow-up
Hoogendoorn et al., 2009 [8]	55	Female	Painful vulvar mass	37 × 36 × 35 mm	Surgical	N/A
Gadde et al., 2011	60	Female	Painless lump	46 mm	Cyst aspiration under CT guidance	N/A
Sava et al., 2012 [11]	59	Female	Incomplete bladder emptying and sharp vaginal pain	N/A	Surgical	N/A
Tan et al., 2012 [4]	69	Female	Painless lump	50 × 30 × 30 mm; 30 × 20 × 30 mm	Observation after open biopsy	—
Ghareeb et al., 2013 [15]	68	Female	Acute urinary retention	44 × 35 × 42 mm	Surgical	No recurrence over a 28-month follow-up
Farag et al., 2014 [3]	61	Female	Difficult micturition and dyspareunia	32 × 30 × 39 mm	Surgical	No recurrence over an 11-month follow-up
	56	Female	Difficult micturition	30 × 38 × 27 mm	Surgical	No recurrence over a 4-month follow-up
Wylie et al., 2014 [2]	69	Male	Pain in the base of penis and scrotum	25 mm	Observation	Size reduction over a 4-year follow-up
Healy et al., 2016 [14]	75	Female	Painless lump	32 mm	Surgical	N/A
Nishisho et al., 2016 [5]	59	Female	Painful vulvar mass	15 × 10 × 10 mm	Observation	Mass resorption after a 48-month follow-up
Strother et al., 2016 [6]	62	Female	Painful vulvar mass	30 × 24 × 32 mm	Surgical	—

CT, computed tomography; MRI, magnetic resonance imaging.

## References

[1] Alguacil-Garcia A., Littman C. D. (1996). Subpubic cartilaginous cyst: report of two cases. *American Journal of Surgical Pathology*.

[2] Wylie K. R., Griffiths J., Pye J., Salim F., Inman R. (2014). A subpubic cartilaginous cyst causing neurological and sexual symptoms in a 69-year-old man. *The Journal of Sexual Medicine*.

[3] Farag F., van der Geest I., Hulsbergen-van de Kaa C., Heesakkers J. (2014). Subpubic cartilaginous pseudocyst: orthopedic feature with urological consequences. *Case Reports in Urology*.

[4] Tan T. J., Wong S. K., Foo L. S. S. (2012). A parasymphyseal pubic cartilaginous cyst masquerading as a chondrosarcoma. *Clinical Radiology*.

[5] Nishisho T., Takao S., Miyagil R., Toki S., Nagamachi A., Sairyo K. (2016). Complete spontaneous regression of a subpubic cartilaginous cyst: a case report. *Journal of Medical Investigation*.

[6] Strother M. C., Weissbart S., Brooks J. S., Smith A. R. (2016). Subpubic cartilaginous cyst–a rare periurethral lesion with implications for surgical approach. *Urology*.

[7] Gadde S., Brisson M., O’Donnell P. (2011). Sub-pubic cartilaginous cyst–diagnostic utility of CT-guided contrast injection. *Skeletal Radiology*.

[8] Hoogendoorn R. W., Kayser H. W. M., Weening J. J., van Geloven A. A. W. (2009). Subpubic cartilaginous cystic lesion presenting as a vulvar mass: a case report. *Journal of Medical Case Reports*.

[9] Bullock R. W., Soares D. P., Shah S. (2009). Subpubic cartilaginous cyst: an unusual cause of a vulval mass. *British Medical Journal Case Reports*.

[10] Martel J. P., Spouge A. R. (2007). Gas-filled parasymphyseal pubic cyst associated with degenerative joint disease. *Skeletal Radiology*.

[11] Sava M. R., Rubin B., Sundaram M. (2012). Subpubic cyst. Subpubic degenerative cyst arising from the fibrocartilage of the pubic symphysis. *Skeletal Radiology*.

[12] Kim C. E., Beasley H. S. (2004). MRI diagnosis of subpubic cartilaginous cyst. *American Journal of Roentgenology*.

[13] Ergun T., Lakadamyali H., Aydin O. (2008). Subpubic cartilaginous cyst: incidental finding detected by abdominopelvic computed tomography. *Radiation Medicine*.

[14] Healy C. F., Pang E., Bahksh S. (2016). Subpubic cartilaginous cyst: a rare sub-clitoral mass. *Journal of Obstetrics and Gynecology Canada*.

[15] Ghareeb G. M., Grabemeyer H., Dietrich E., Heisler C. A. (2013). Subpubic cartilaginous cyst presenting as acute urinary retention: a report and review of the literature. *Female Pelvic Medicine and Reconstructive Surgery*.

[16] Judson P. L., Ivy J. J., Zwolak P., Manivel J. C., Clohisy D. R. (2009). A suspicious vulvar mass diagnosed as a subpubic cartilaginous cyst. *Archives of Gynecology and Obstetrics*.

[17] Becker I., Woodley S. J., Stringer M. D. (2010). The adult human pubic symphysis: a systematic review. *Journal of Anatomy*.

